# Effects of priority on strain-level composition of the honey bee gut community

**DOI:** 10.1128/aem.00828-25

**Published:** 2025-07-31

**Authors:** Korin Rex Jones, Yulin Song, Sabrina Sakurako Rinaldi, Nancy A. Moran

**Affiliations:** 1 Department of Integrative Biology, University of Texas at Austin 171878, , Austin, Texas, USA; Norwegian University of Life Sciences, Ås, Norway

**Keywords:** symbiont, coexistence, microbiome, bacteria, bee, honey bee, *Apis mellifera*, type VI secretion system, community ecology, microbial ecology

## Abstract

Host-associated microbiomes are complex communities shaped by interactions between members. The type VI secretion system (T6SS), among other bacterial weapons, allows gram-negative bacteria to deliver toxic effectors into competitors. In this study, we investigated the impact of differential colonization timing on the competitive advantage associated with T6SS possession using *Snodgrassella alvi*, a core symbiont of the honey bee gut microbiome. Following a timeline based on the natural establishment window of the gut microbiome, we sequentially inoculated newly emerged bees with fluorescently labeled strains that differed in presence of the T6SS-1. When inoculated simultaneously, the T6SS-1-possessing strain (wkB2) consistently excluded the T6SS-1-lacking strain (wkB332); however, when given a 5-day advantage, the second strain was consistently excluded regardless of strain identities. With a 1-day advantage, the effect of priority was weakened, but wkB332 was able to persist following introduction of wkB2. Utilizing a wkB2 T6SS-1 knockout strain, we repeated our 24 hour priority experiments and found that the T6SS-1 contributes to invasion outcomes along with other mechanisms of competition. Through fluorescent microscopy, we explored how coexisting strains in these experimental scenarios organize spatially within the bee ileum. Our results demonstrate that colonization timing can have lasting consequences for strain composition of the established microbiome. These findings illustrate the influence of stochastic processes in microbial community assembly and emphasize that differences in colonization timing may alter competitive outcomes between taxa, impacting taxon coexistence.

**IMPORTANCE:**

The bacterial gut communities of honey bees possess considerable strain-level diversity between hives, between individual bees, and within individual bees. However, the factors underlying strain coexistence are unclear. Here, we provide support for timing of colonization, or priority effects, as one factor driving this strain-level diversity. Our results show that priority inoculation can prevent colonization by subsequent competing bacterial strains and mitigate advantages conferred through bacterial weaponry. Further, a brief window of priority can facilitate the coexistence of strongly and weakly competitive strains within single bees. These results add to our understanding of the impacts of priority effects in host-associated microbial communities. Such an understanding can aid the development of future probiotic strategies aimed at improving honey bee health.

## INTRODUCTION

Host-associated microbiomes are complex communities that consist of interacting species of microorganisms. The ultimate structure of these communities, specifically which microbes are present and in what proportions, can have consequences for host health and development (1–4). Microbiome composition has been shown to impact host obesity (5), disease susceptibility (6, 7), vulnerability to toxins (4, 8), and behavior (9, 10). Because of this structure-function relationship, understanding the processes governing how communities assemble continues to be an important goal in microbial ecology (11, 12).

Broadly, the assembly of ecological communities is understood to be governed by four ecological processes: selection, dispersal, speciation, and drift (13). Among different systems and conditions, the relative influence of each of these factors will fluctuate (14). Even among equivalent habitats, stochasticity may result in differences in the microbiome community composition (15, 16). For example, differences in the arrival order of bacterial colonists can sometimes impact microbiome composition, commonly described as priority effects (17–19). Early arriving species may dominate resources (niche preemption) or modify the environment (niche modification) in a way that hinders the colonization ability of subsequent species (12, 20, 21). In flower nectar, for example, early arriving *Acinetobacter* can lower the pH, preventing future colonization by yeasts (21). Because priority effects are expected to occur in situations where fitness differences between competing taxa are low and stabilizing forces are absent (22), they may be especially prominent in strain-strain competition during colonization.

In addition to stochastic processes, deterministic processes also influence the microbiome structure (23, 24). Bacteria can possess a diverse array of weaponry, providing advantages for certain strains over others (25, 26). The type VI secretion system (T6SS) is one such mechanism, capable of delivering effector proteins into neighboring cells in a contact-dependent manner, with individual strains possessing immunity mechanisms against their own effectors (27). Initially implicated in pathogenesis, the T6SS has since been found to determine competitive outcomes within and among bacterial species (28, 29). For example, an *in planta* coinfection assay found that the soil bacterium *Agrobacterium tumefaciens* utilized the competitive advantage provided by its T6SS to attack and kill *Pseudomonas aeruginosa* cells (30). Similarly, experimental inoculations of *Vibrio fischeri*, an essential symbiont that occupies the light organ of the Hawaiian bobtail squid (*Euprymna scolopes*), have demonstrated that competing strains utilize the T6SS in strain-strain competition during colonization of their squid hosts (31). In T6SS-mediated antagonism, the more abundant strain has an advantage as it possesses immunity mechanisms against its own weaponry, resulting in positive feedback.

The gut microbiome of honey bees (*Apis mellifera*) is a simple, conserved community of bacteria largely dominated by a few key members (*Bartonella, Bifidobacterium*, *Bombilactobacillus* [formerly “Firm-4”], *Commensalibacter, Frischella, Gilliamella, Lactobacillus* nr. *melliventris* [formerly “Firm-5”], and *Snodgrassella*) (32, 33). Prior research on this community suggests that both stochasticity in dispersal and deterministic competitive processes likely influence community composition (34–36). Within the genera *Snodgrassella* and *Gilliamella*, which together form a densely packed biofilm in the ileum region (37), different approaches have shown strain-level variation among gut communities of hive mates that may be driven by stochastic processes such as priority effects (34, 38). Both *Snodgrassella* and *Gilliamella* include strains that encode T6SSs and a diverse array of associated effectors (39, 40).

*Snodgrassella alvi* is a primary colonist that characteristically forms a biofilm on the ileum wall, where it may interact with the host immune system and influence the colonization of other taxa (33, 41). The *S. alvi* type strain wkB2 possesses two T6SSs. T6SS-1 facilitates intraspecific competition, and T6SS-2 promotes colonization of the host (35). Other strains of this species can possess one, both, or neither of these T6SSs (39, 40).

Prior studies showed that when strains colonize a bee ileum simultaneously, possession of the T6SS-1 and corresponding immunity gene determines which strain dominates (35). It is thus unclear how *S. alvi* strains that lack immunity to T6SS-1 effectors persist in populations of host bees. Stochasticity in colonization timing within individual bees, which facilitate priority effects, may be one explanation (34).

In the current study, we inoculated newly emerged, microbiota-deprived bees with one of three fluorescently tagged (42) strains of *S. alvi* in a pairwise sequence and included delays between the introduction of the first and second strain to better understand colonization dynamics in the honey bee gut. We chose two naturally occurring strains that differ in their encoded T6SS genes; wkB2 (T6SS-1 and T6SS-2) and wkB332 (T6SS-2 only). We also included a T6SS-1 knockout of wkB2 to disentangle the impact of T6SS antagonism from other contrasting strain characteristics. We hypothesized that if a strain vulnerable to T6SS attack were to be established prior to the introduction of a T6SS-possessing strain, the temporal advantage would outweigh its vulnerability and facilitate its persistence.

## MATERIALS AND METHODS

### Bacterial cultures

*S. alvi* strains wkB2 and wkB332 were previously isolated from guts of *A. mellifera* and were used in a previous study on strain interactions (35). For the wkB2 T6SS-1 knockout strain, we used wkB2 ΔtssE-1, which was constructed in a previous study (35).

Strains were cultured on Columbia agar supplemented with 5% defibrinated sheep blood (hereafter CBA). *Escherichia coli* cultures were grown on LB agar, LB broth, or CBA. The culture medium was supplemented as needed with 0.3 mM diaminopimelic acid (DAP), 12.5 µg/mL tetracycline (tet), 50 µg/mL kanamycin (kan), and/or 60 µg/mL spectinomycin (spec). Plated cultures were grown in a 5% CO2 incubator at 35°C.

All strains used in this experiment can be viewed in Table 1.

**TABLE 1:** Table of experimental strains. Non-bolded strains were utilized to create the bolded strains used in this experiment

Strain ID	Fluorescent marker	Antibiotic resistance marker(s)	Species	Reason for inclusion	Used in experiment no.
MFD-GFP	GFP	Kanamycin	*Escherichia coli*	Transformation donor strain	N/A^*b*^
MFD-RCP	RCP	Kanamycin	*Escherichia coli*	Transformation donor strain	N/A
wkB2	None	Tetracycline	*Snodgrassella alvi*	Parent strain	N/A
wkB332	None	Tetracycline	*Snodgrassella alvi*	Parent strain	N/A
wkB2-ΔtssE	None	Spectinomycin	*Snodgrassella alvi*	Mutant parent strain	N/A
**wkB2-RCP^*a*^**	RCP	Kanamycin, tetracycline	*Snodgrassella alvi*	Experimental strain	1,2,3
**wkB2-GFP**	GFP	Kanamycin, tetracycline	*Snodgrassella alvi*	Experimental strain	1,2,4
**wkB332-RCP**	RCP	Kanamycin, tetracycline	*Snodgrassella alvi*	Experimental strain	1,2,4
**wkB332-GFP**	GFP	Kanamycin, tetracycline	*Snodgrassella alvi*	Experimental strain	1,2,3
**wkB2-ΔtssE-RCP**	RCP	Kanamycin, tetracycline, spectinomycin	*Snodgrassella alvi*	Experimental strain	3
**wkB2-ΔtssE-GFP**	GFP	Kanamycin, tetracycline, spectinomycin	*Snodgrassella alvi*	Experimental strain	4

^*a*^ Boldface indicates strains that were used in our experiments.

^*b*^ N/A indicates that these strains were not used in any of the experiments. These strains were only used to create our experimental strains.

### Strain transformation

All strains used in this study were transformed using the Pathfinder plasmid system, described in Elston et al. (42). Briefly, cultures of *E. coli* MFDpir containing GFP (pSL1-GFP) or RCP (red chromoprotein; pSL1) plasmids were started on LB agar supplemented with kan and DAP. Cultures of *S. alvi* were simultaneously started on CBA. The next day, single colonies from the MFDpir strains were placed in LB broth supplemented with kan and DAP overnight. The following day, *S. alvi* strains were scraped and suspended in 500 µL PBS. *S. alvi* suspensions and 500 µL of MFDpir strains were spun down at 14,000 rpm for 3 minutes. The liquid was replaced with a fresh 500 µL aliquot of PBS and vortexed to resuspend the pellet. After repeating this a second time, the OD of each suspension was measured, and strains were mixed at a ratio of 1:10 donor to recipient. One hundred microliters of each mixture was spot-plated on CBA plates supplemented with DAP and placed in an incubator overnight. The following day, cultures were scraped and resuspended in 500 µL PBS. Full and 1:10 dilutions of these resuspended cultures were then plated onto kan-/tet- or kan-/tet-/spec-supplemented CBA plates. Tetracycline was used on these selective plates as wkB2 and wkB332 have natural tetracycline resistance. Single fluorescent colonies were then picked and plated on kan/tet CBA plates. GFP and RCP variants for each strain were created and stored in 25% glycerol at −80°C.

### Inoculum preparation

Stock cultures of *S. alvi* strains were plated on kan CBA plates and incubated for 2 days. Single colonies were then picked from these plates, struck onto new kan CBA plates, and incubated for 2 days. Plates were then scraped and resuspended in a 1.5 mL microcentrifuge tube containing 500 µL PBS. Optical densities of these suspensions were measured in a BioSpectrometer (Eppendorf, CT), and the volume of culture needed to form 1 OD of solution was placed in a new microcentrifuge tube and spun-down at 14,000 rpm to separate the bacterial pellet from the supernatant. After centrifugation, the supernatant was discarded. Bacteria were resuspended in 500 µL PBS and 500 µL 1:1 sterile sucrose:water solution. The sugar/water solution was added at the time of hand-feeding to minimize potential cell death caused by the solution.

### Priority inoculation experiments

**Fig 1:**
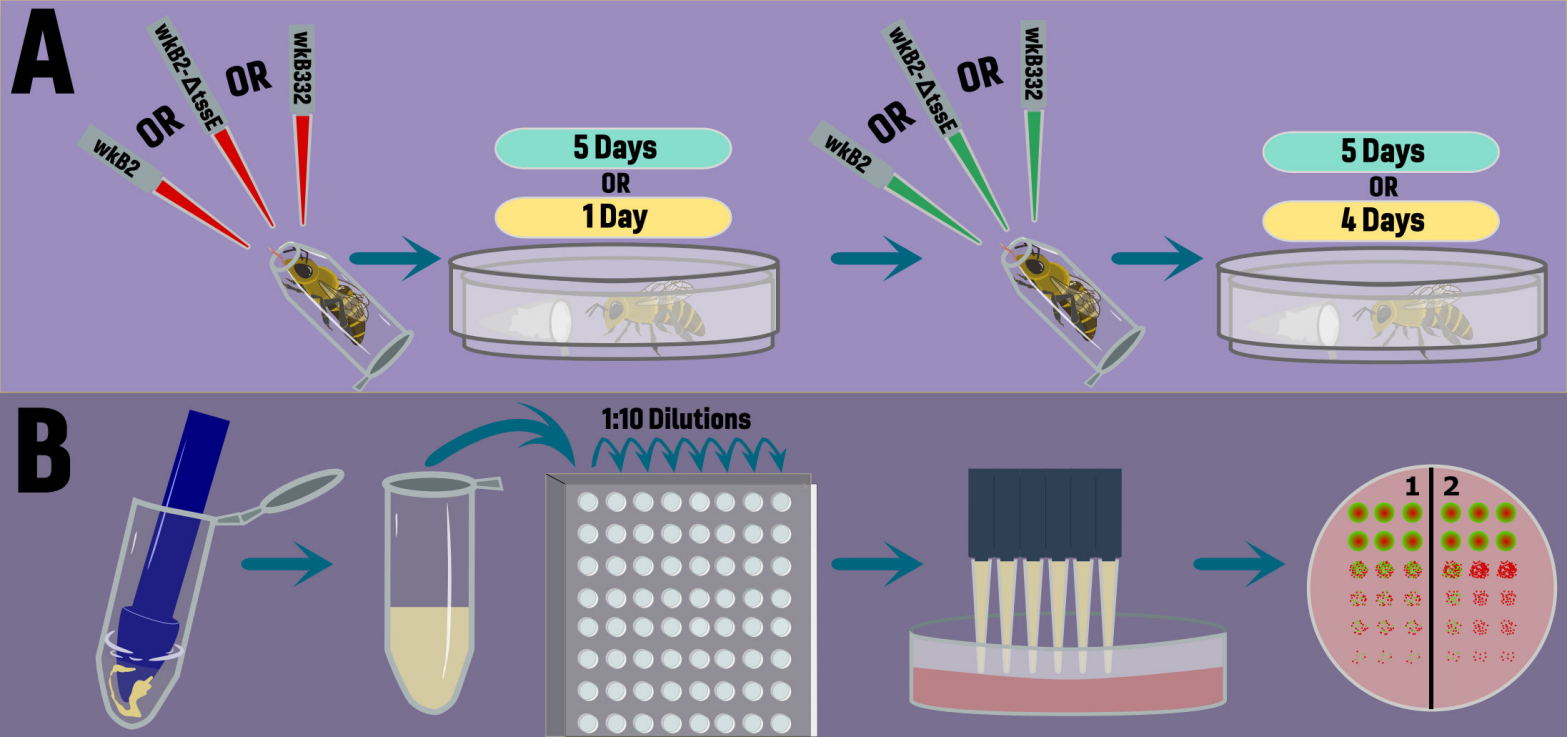
Concept diagram explaining the experimental design. (**A**) Newly emerged, microbiota-deprived honey bees were sequentially inoculated with fluorescently tagged strains of *S. alvi* (wild-type wkB2, wkB2 ΔtssE-1knockout, or wild-type wkB332) over two experimental timelines. Bees were either inoculated with a secondary strain after 5 days, followed by 5 days of incubation or after 1 day, followed by 4 days of inoculation. (**B**) Once the experiment was completed, bees were dissected, their guts were homogenized, and dilution series were conducted in 96-well plates. These dilutions were then plated on Columbia blood agar plates, and CFUs were identified and counted based on fluorescence.

To obtain microbiota-deprived newly emerged worker bees of *A. mellifera*, we followed an established protocol (see [35, 35]) with minor modifications. Briefly, brood frames were collected from two hives located on the University of Texas at Austin campus. Pupae in late development, characterized based on body and eye pigmentation, were extracted from frames using sterile forceps. Collected pupae were then placed into sterilized plastic containers lined with Kimwipes and supplied with sterile 1:1 sucrose:water solution. Pupae were kept in a 35֯°C incubator with ~60% relative humidity to mimic hive conditions. After a few days, we transferred newly emerged, microbiota-deprived worker bees to sterile feeding tubes (0.5 mL microcentrifuge tubes with the ends cut off) (Fig. 1A). Bees were starved for ~2 hours and then hand-fed a 5 µL treatment inoculum. This inoculum contained a sucrose solution composed of either a single strain or an evenly proportioned mixture of two strains. After feeding, bees were placed into individual polypropylene Petri dishes (Eisco, NY) containing a 0.5 mL microcentrifuge tube filled with sterile 1:1 sucrose:water solution. After the priority period had elapsed for the initial strain, bees were briefly chilled to restrict movement and then placed in new sterile feeding tubes and starved for ~2 hours, before being fed the second inoculum and returned to their individual Petri dishes. Bees were kept in the incubator at 35֯°C until the end of the experiment.

To assess colonization outcomes at the end of the incubation period, bees were chilled and their guts dissected with flame-sterilized forceps. Extracted guts were homogenized in 100 µL PBS using a BioVortexer (BioSpec, OK) and sterilized polypropylene pestles for 30 s (Fig. 1B). Pestles were then rinsed with 400 µL of PBS, bringing the total volume to 500 µL, and stored on ice. Twenty microliters of each sample was then used to prepare 10-fold serial dilutions. Ten microliters of each dilution was plated in triplicate on kan-supplemented CBA plates and incubated for three days. Gut homogenates for microbiota-deprived bees were plated on both CBA and kan supplemented CBA. Colony-forming units (CFUs) were then counted for each sample from the dilution with the largest number of countable colonies (distinguishable individual colonies).

We measured colonization success as (i) invasion success and (ii) relative strain abundance. For invasion success, we scored whether the second strain was present at any level. For relative strain abundance, we used the CFU data to calculate relative abundances of the two strains.

### Individual experimental design

Our experimental design consisted of two types of priority inoculation experiments (Fig. 1A). First, we inoculated newly emerged, microbiota-deprived bees with a single strain of *S. alvi*, allowed 5 days for that strain to colonize, introduced the second strain, and then waited an additional 5 days before concluding the experiment and performing dissections for CFU counts (5d/5d). The normal timeline for establishment of the honey bee microbiome is ~4 days (43); therefore, this experimental design gives an insight into a strain attempting to invade a “fully colonized” ileum. Second, we employed a design in which we restricted the initial strain to only 1 day of priority before the second strain was introduced and then 4 additional days for the bacteria to colonize the host bee (1d/4d) (Fig. 1A). We also included a mixed-treatment design in which strains were introduced simultaneously on day 1. These samples were dissected on the same day as the other bees in the 1d/4d experiment. With this strategy, we sought to more closely mimic natural scenarios that may take place in the hive environment by keeping our timeline within the natural period of microbiome establishment (43). We performed four experiments sequentially: (i) 5d/5d with both fluorescent markers for each wild-type (WT) strain, (ii) 1d/4d with both fluorescent markers for each WT strain, (iii) 1d/4d with one set of fluorescent markers for our two WT and T6SS knockout strains, and (iv) 1d/4d with swapped fluorescent markers for our two WT strains and T6SS knockout strain (e.g., RCP strains in experiment 3 expressed GFP in experiment 4. Although the markers have no evident effect on growth *in vivo*, we were able to eliminate the possibility that differences were due to a marker effect by reversing the makers. Experiments 1 and 2 used bees from a single hive and are analyzed individually. Experiments 3 and 4 were performed sequentially using bees from a second hive. Data from experiments 3 and 4 were analyzed as a single data set as the only difference between these experiments was the assignment of a fluorescent marker for each strain. All data and code used in this study are available in the supplemental data set.

### Imaging honey bee ileum with fluorescent microscopy

Honey bees were raised and inoculated as above. Three bees from each treatment in experiment 3 (1d/4d) were selected and processed for imaging on the final day of the experiment. Whole guts were dissected and mounted with PBS onto glass slides under a Leica MDG41 stereoscope, and Malpighian tubules were separated from view of the ileum. The ileums of dissected guts were then imaged using Nikon NIS-Elements software (AR 5.30.05 Build 1559) and a Nikon Eclipse TE2000-U inverted fluorescent microscope in GFP and RCP channels to visualize bacterial colonization.

### Statistical analysis

Statistical analyses were performed in the R v4.3.1 environment (44) via R studio (45) (build 524).

To understand if inoculation order influenced invasion success, we scored each bee as “invaded” or “not invaded” according to whether the second strain was detectable at the sampling time. These data were analyzed using chi-squared tests.

To determine if inoculation order impacted strain relative abundance, we performed Wilcoxon rank-sum tests on relative abundances calculated from CFU counts.

Plots were created using the ggplot (3.4.2) (46), ggpubr (v0.6.0) (47), scales (v1.2.1) (48), and phyloseq (1.44.0) (49) packages.

## RESULTS

**Fig 2:**
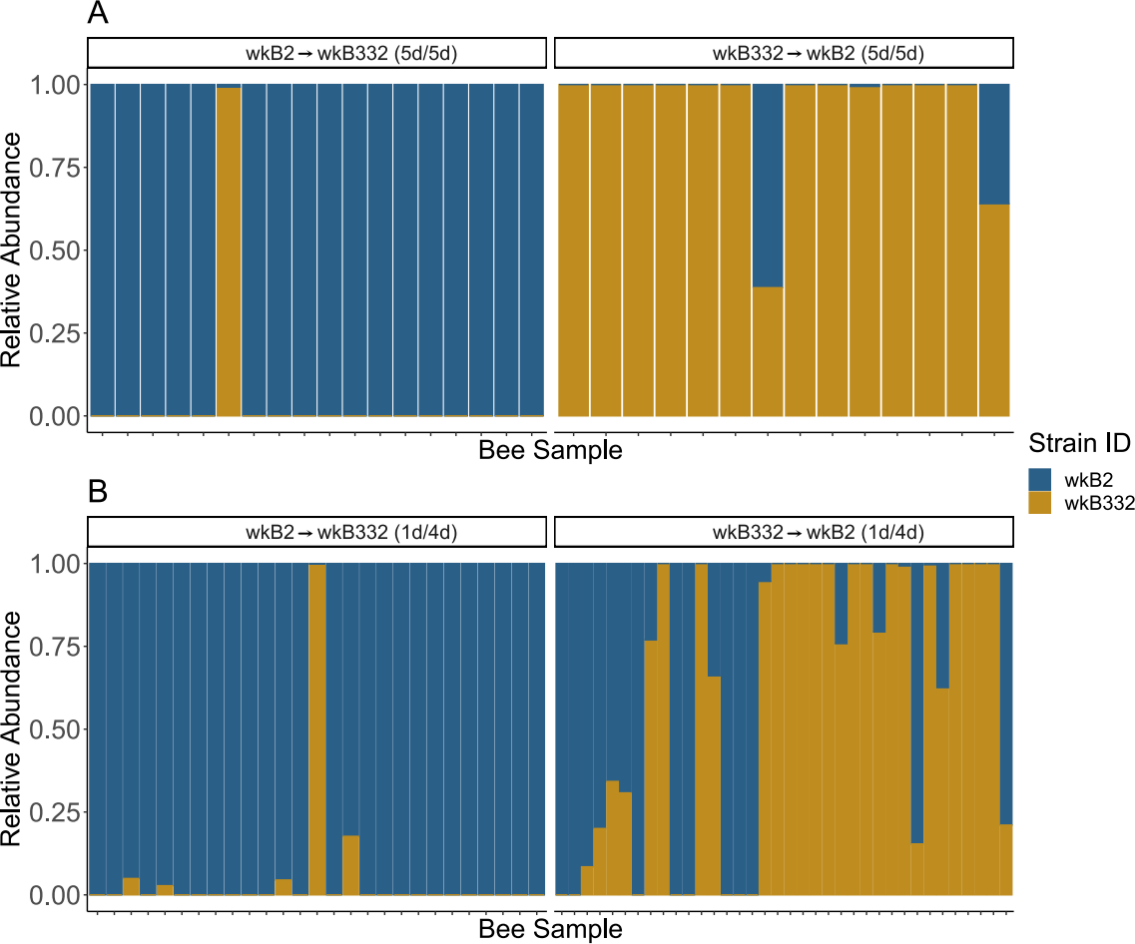
Relative abundance plots based on CFU counts of *S. alvi* indicating proportions of wild-type wkB2 and wkB332 present during (**A**) 5 day/5 day or (**B**) 1 day/4 day experimental timelines based on the inoculation order.

Experimentally inoculating microbiota-deprived, newly emerged honey bees with one strain of *S. alvi* and a second strain 5 days later led to strong priority effects (5d/5d) (Fig. 2). Samples were dominated by the initial strain. Which strain arrived first (wkB2 or wkB332) did not affect the ability of the second strain to invade (Chi-sq = 1.81, *P*-value = 0.18) (Table 2). Further, initial strain identity did not affect relative abundances (Wilcoxon rank sum *P*-value = 0.23) (Fig. 2A). The initial strain averaged at least 87% at the time of sampling.

**TABLE 2:** Ability of strains to invade in experimental inoculations

Inoculation order		Invaded^*a*^	Not invaded	Total	Chi-squared value	*P* value
5 day/5 day experiment						
wkB2→wkB332		1	17	18	1.81	0.18
wkB332→wkB2		3	11	14		
1 day/4 day experiment						
wkB2→wkB332		5	22	27	11.43	0.0007
wkB332→wkB2		22	14	36		

^*a*^ Invaded means that the second strain was present at any level at the time of sampling.

Restructuring our inoculation timeline to be within the normal window of microbiome establishment in honey bees, we examined priority effects when a strain is given only 1 day of priority (1d/4d). In experiment 2, we used 1d/4d and found that invasion success of the second strain depended on initial strain identity, with wkB2 being invaded less often (Chi-sq = 11.43, *P*-value = 0.0007) (Table 2). Likewise, percentages of the secondary strain were lower when wkB2 was the initial strain (Wilcoxon rank sum *P*-value = 0.00024) (Fig. 2B).

To understand the potential role of T6SS-1 in these colonization outcomes, we performed a 1d/4d experiment in which we included a T6SS-1 knockout mutant (wkB2 ΔtssE-1). When given priority, wkB2 WT was far more successful at preventing invasion by wkB332 than was wkB2 ΔtssE-1 (Chi-sq = 12.44, *P*-value = 0.00042). Similarly, wkB332 was invaded by wkB2 WT more often than by the wkB2 ΔtssE-1knockout strain (Chi-sq = 5.07, *P*-value = 0.024) (Table 3).

**TABLE 3:** Ability of strains to invade in experimental inoculations in the 1d/4d experiment that includes wkB2 with inactivated T6SS-1

Inoculation order	Invaded^*a*^	Not invaded	Total	Chi-squared value	*P* value
wkB2→wkB332	0	33	33	12.44	0.0004
ΔtssE→wkB332	9	19	28		
wkB332→wkB2	34	4	38	5.07	0.0244
wkB332→ΔtssE	26	12	38		

^*a*^ Invaded means that the second strain was present at any level at the time of sampling.

**Fig 3:**
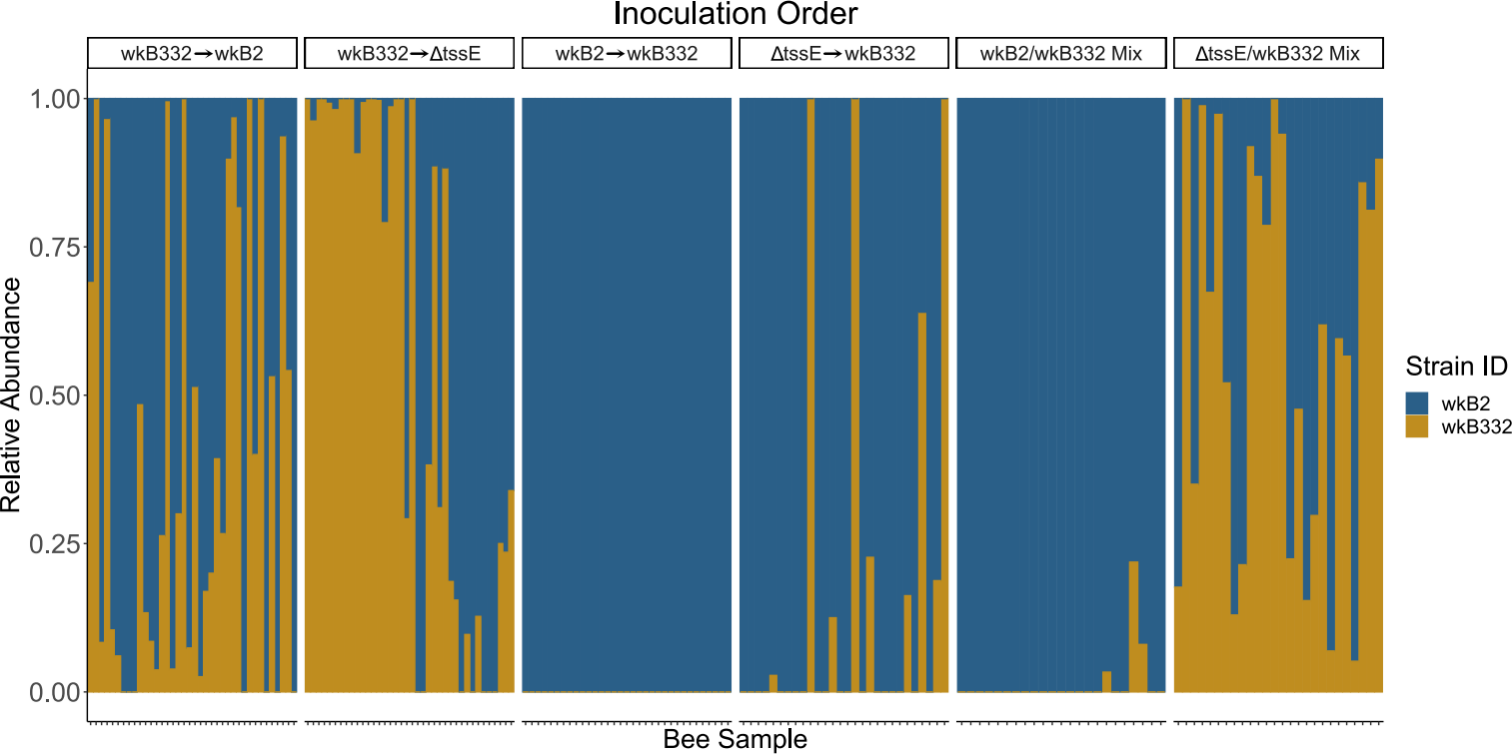
Relative abundance plot based on CFU counts of *S. alvi* indicating proportions of wild-type wkB2, wkB2 ΔtssE-1, and wild-type wkB332 based on experimental inoculation order. “Mix” refers to simultaneous inoculation of the two strains.

We next evaluated the overall impact of priority versus simultaneous inoculation on strain proportional abundances when wkB332 is paired with either wkB2 WT or wkB2-ΔtssE-1. When wkB332 and wkB2 WT were paired together, wkB2 WT had a greater proportional advantage when it had a 1d priority versus when introduced simultaneously with wkB332 (Wilcoxon rank sum test. *P*-value = 0.037). However, wkB2 WT dominated even in the latter case; only 3 of the 23 simultaneously inoculated communities contained wkB332 (Fig. 3). For wkB332, we also saw an increase in the relative abundance with 1d priority compared to simultaneous inoculations when the second strain was wkB2 WT (Wilcoxon Rank Sum Test *P*-value = 3.1e-07) (Fig. 3). In pairings of wkB2-ΔtssE-1 with wkB332, introducing wkB2-ΔtssE-1 first led to an increase in its relative abundance compared to simultaneous inoculation (Wilcoxon rank sum test *P*-value = 5.6e-06) (Fig. 3). For wkB332, however, we did not see greater relative abundance when introduced first compared to simultaneous inoculations when the second strain was wkB2-ΔtssE-1 (Wilcoxon rank sum test *P* = 0.45) (Fig. 3).

To understand the potential influence of the T6SS on competitive outcomes between our isolates during our inoculation experiments, we analyzed differences in abundances of wkB332 between priority treatments. When wkB332 was second, it showed a higher relative abundance with wkB2-ΔtssE-1 than with wkB2 WT (Wilcoxon rank sum test *P*-value = 0.00052) (Fig. 3). When wkB332 was first, it had a greater abundance with wkB2-ΔtssE-1 than with wkB2 WT (Wilcoxon sum rank test *P*-value = 0.042) (Fig. 3).

**Fig 4:**
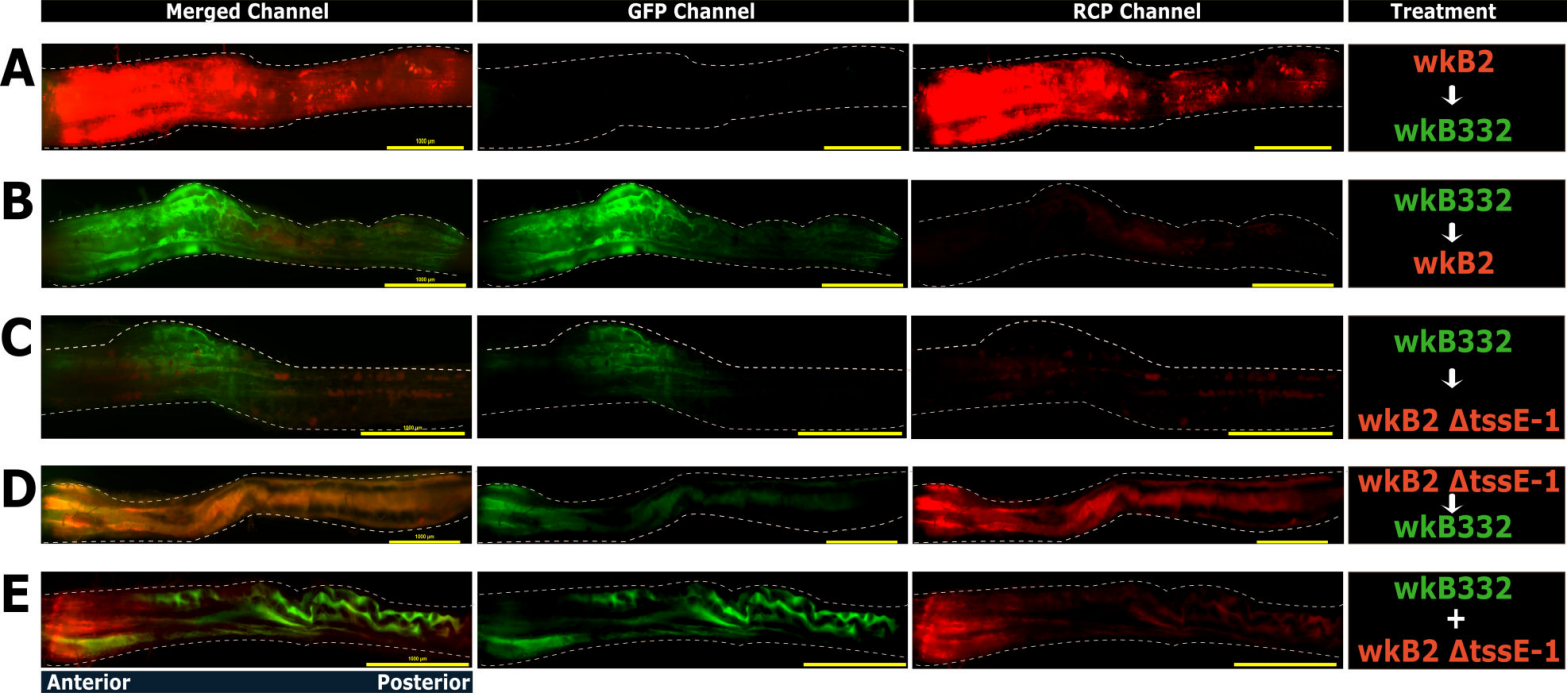
Images of honey bee gut colonization treatments, generated through fluorescent microscopy. The image includes only the ileum section of the gut and is oriented from pylorus-adjacent to rectum-adjacent, left to right. Wild-type wkB332 is colored green in these images, while wild-type wkB2 and wkB2 ΔtssE-1 are colored red. The delay between inoculations was 1 day, with sampling 4 days later. (**A**) wkB2 WT followed by wkB332; (**B**) wkB332 followed by wkB2 WT; (**C**) wkB332 followed by wkB2 ΔtssE-1; (**D**) wkB2 ΔtssE-1 followed by wkB332; (**E**) wkB332 simultaneously inoculated with wkB2 ΔtssE-1.

To better understand scenarios that implied coexistence between strains, we examined the spatial context of these outcomes by imaging intact ileums from a subset of bees from each treatment using fluorescence microscopy, following a 1d/4d inoculation sequence. We observed a general preference of all strains for the anterior end of the ileum, with the densest fluorescence observed near the pylorus (Fig. 4, alternate color scheme in Fig. S1), potentially reflecting local differences in nutrient availability, pH, or other factors. When wkB2 WT was first, it dominated, with wkB332 not detectable, consistent with previous experiments. When wkB332 had priority, it formed large, continuous patches of colonization with considerably smaller patches of wkB2/wkB2-ΔtssE-1 at both the anterior and posterior ends of the ileum (Fig. 4A and B). When wkB2-ΔtssE-1 was introduced first, we saw considerable overlap in fluorescent signals between strains (Fig. 4C). We observed limited overlap between wkB332 and wkB2-ΔtssE-1 when inoculated simultaneously (Fig. 4D).

## DISCUSSION

Microbiome composition reflects the interplay between stochastic and deterministic factors. Here, we demonstrate the strengths of the honey bee gut microbiome as a system for understanding that interplay. Additionally, this work provides support for priority effects as a mechanism by which weakly competitive symbiont strains might persist in the presence of more competitive strains. The relationship between *S. alvi* strains wkB2 and wkB332 is reaffirmed through our experiments, confirming that the T6SS-1 system of wkB2 contributes to its ability to routinely outcompete wkB332 in directly competitive scenarios (31, 35). We build on this understanding by showing that temporal advantages can support the persistence of wkB332 in the presence of wkB2, with the strength of these advantages dependent on the presence of T6SS-1.

Priority effects are thought to affect compositional outcomes across systems in community ecology. The term has been used as early as 1987 to describe the ability of dominant plant species to prevent the establishment of late-emerging competitors (50); conversely, early arriving taxa may facilitate subsequent colonists (51). In the case of intraspecific competition between strains of bacteria, the early arriving taxa can completely or partially exclude subsequent taxa (18, 52). In our experimental design, we utilized two lengths of delayed inoculations to better understand how time might influence these outcomes. For our two isolates, we saw consistent exclusion of invaders at our longer delay period of 5 days; however, only wkB2 consistently repelled invasion in our 1-day delay experiments. Honey bees establish their gut microbiomes by approximately the fourth day post-emergence (43). Our results suggest that, after this period of establishment, strain proportions of *S. alvi* within the gut become relatively stable.

Because honey bees do not leave the hive until ~21 days post-emergence (53), the inability of late-arriving strains to colonize might exclude *S. alvi* strains encountered outside the hive, including *Snodgrassella* strains associated with other bee species foraging at the same flowers. Thus, the delay in exposure may contribute to the host specificity documented for *Snodgrassella* (54, 55), although host-specific regulatory mechanisms also contribute (56, 57).

In our 1-day delay experiments, we saw distinct outcomes based on strain identity and the presence or absence of a working T6SS-1 system. When the T6SS-1 possessing strain wkB2 was inoculated first, wkB332 was greatly inhibited. Disabling the T6SS-1 increased the ability of wkB332 to invade, indicating that the T6SS-1 helps exclude later arriving *S. alvi* strains. The *S. alvi* T6SS-1 system was also beneficial when invading an established *S. alvi* population as *S. alvi* wkB2 was more able to invade wkB332 when the T6SS-1 was active. As suggested by an earlier study (35), other mechanisms affect competitive success in these strains, as even the wkB2 knockout achieves a higher relative abundance than wkB332, regardless of the order of arrival.

Because the T6SS action is contact-dependent, the advantage it confers to the more abundant strain is confined to its immediate surroundings. Potentially, different strains, each with distinct toxin-immunity systems, could establish at different locations in the uncolonized gut and ultimately dominate within local patches. If so, an established community within a single bee gut might contain multiple strains, each confined to distinct regions. Colonization patterns of fluorescently tagged strains gave some evidence of such patchiness; however, our images of intact guts do not resolve fine-scale spatial organization. Depending on whether the mechanism of antagonism is localized, as for T6SS, or more dispersed, as for diffusible toxins such as bacteriocins (58), different outcomes might be expected.

A goal of our study was to better understand how strain diversity is maintained despite variation in antagonism mechanisms that enable some strains to dominate over others. Differences in resource use, or ecological niche, could also contribute to coexistence, as appears to be the case for closely related *Gilliamella* species within honey bee guts (55). Compared to *Gilliamella*, *S. alvi* strains vary less in gene content*,* and a study on metabolic requirements of *S. alvi* showed that strains are generally similar in use of substrates within the gut (59). However, we cannot rule out some contribution of niche differences to the maintenance of *S. alvi* strain diversity.

Overall, our results provide evidence of priority effects in the establishment of the honeybee microbiome. Potentially, these effects help explain the persistence of multiple strains of *S. alvi* in populations of honey bees (34). We found that the magnitude of priority effects depends on the timing of arrival of different strains, which, in the context of the honey bee life cycle, may help enforce host specificity since newly emerged bees would always encounter strains from their own species first. We confirm the previously established idea that competitive outcomes between T6SS-possessing and vulnerable strains operate in a deterministic fashion (31, 35). Our findings show that stochasticity in which strain colonizes first can impact these outcomes and can mitigate T6SS-derived advantages. This study joins a growing body of research providing experimental evidence of priority effects within microbial communities and prompts further exploration into the interplay of deterministic and stochastic processes in determining the microbiome composition in hosts.

## SUPPLEMENTAL MATERIAL

Figure S1 aem.00828-25-s0001.pdf Figure 4 with an altered color scheme.

Supplemental text aem.00828-25-s0002.txt Code used for data analysis.

Supplemental data set aem.00828-25-s0003.xls Instructions for interpreting the treatment categories, and abundance and sample data files mentioned in the supplemental text file.

ASM does not own the copyrights to Supplemental Material that may be linked to, or accessed through, an article. The authors have granted ASM a non-exclusive, world-wide license to publish the Supplemental Material files. Please contact the corresponding author directly for reuse.
