# Supplementary material for: Effects of priority on strain-level composition of the honey bee gut community
Source: Appl Environ Microbiol. 2025 Jul 31;91(8):e00828-25. doi: 10.1128/aem.00828-25 (PMC12366320; doi:10.1128/aem.00828-25)
Supplement: Figure S1 — Figure 4 with an altered color scheme. [file aem.00828-25-s0001.pdf]

Supplemental Figure to Jones et al.: “Effects of priority on strain-level composition of the honey bee gut community”

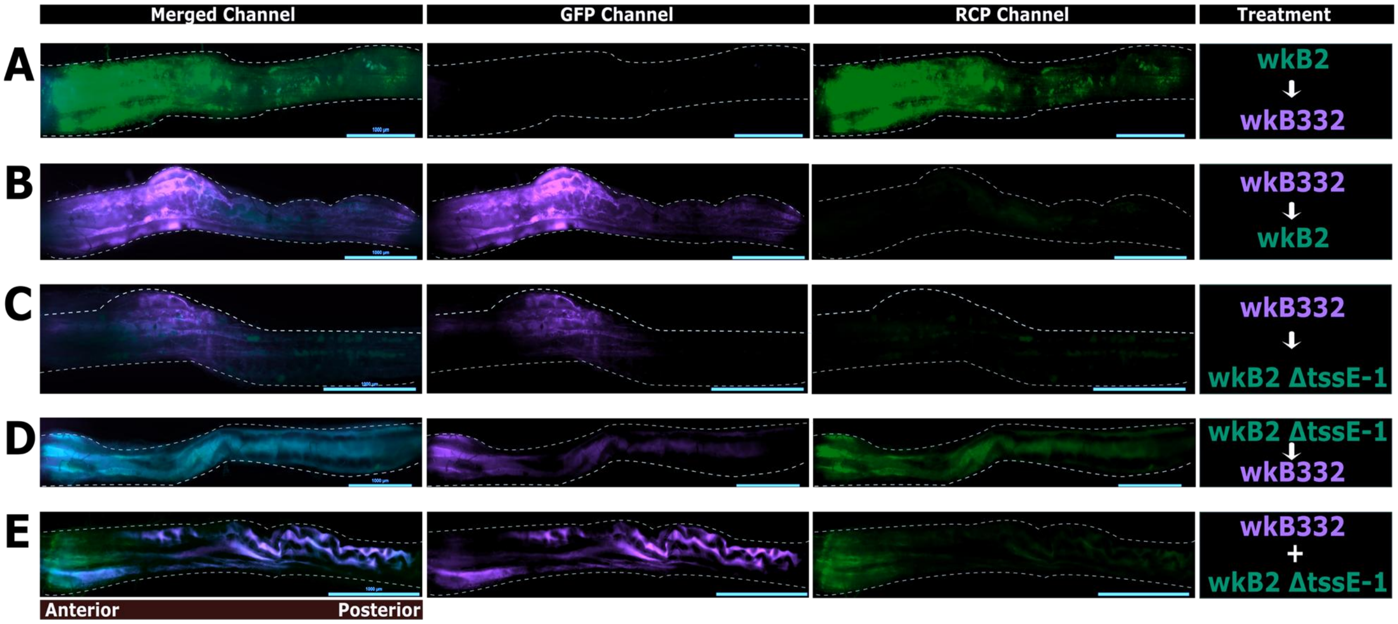

Fig. S1: This figure is the same as Figure 4 with an altered color scheme. Images of honey bee gut colonization treatments, generated through fluorescent microscopy. The image includes only the ileum section of the gut and is oriented from pylorus-adjacent to rectum-adjacent, left to right. Wild type wkB332 is colored green in these images, while wild type wkB2 and wkB2 ΔtssE-1 are colored red. The delay between inoculations was 1 d, with sampling 4 d later. **A)** wkB2 WT followed by wkB332, **B)** wkB332 followed by wkB2 WT, **C)** wkB332 followed by wkB2 ΔtssE-1, **D)** wkB2 ΔtssE-1 followed by wkB332, **E)** wkB332 simultaneously inoculated with wkB2 ΔtssE-1.
